# Calibrationless Parallel Magnetic Resonance Imaging: A Joint Sparsity Model

**DOI:** 10.3390/s131216714

**Published:** 2013-12-05

**Authors:** Angshul Majumdar, Kunal Narayan Chaudhury, Rabab Ward

**Affiliations:** 1 Department of Electrical and Computer Engineering, University of British Columbia, Vancouver, BC V6T 1Z4, Canada; E-Mail: rababw@ece.ubc.ca; 2 Program in Applied and Computational Mathematics (PACM), Princeton University, Princeton, NJ 08544, USA; E-Mail: kchaudhu@math.princeton.edu

**Keywords:** compressed sensing, magnetic resonance imaging, optimization

## Abstract

State-of-the-art parallel MRI techniques either explicitly or implicitly require certain parameters to be estimated, e.g., the sensitivity map for SENSE, SMASH and interpolation weights for GRAPPA, SPIRiT. Thus all these techniques are sensitive to the calibration (parameter estimation) stage. In this work, we have proposed a parallel MRI technique that does not require any calibration but yields reconstruction results that are at par with (or even better than) state-of-the-art methods in parallel MRI. Our proposed method required solving non-convex analysis and synthesis prior joint-sparsity problems. This work also derives the algorithms for solving them. Experimental validation was carried out on two datasets—eight channel brain and eight channel Shepp-Logan phantom. Two sampling methods were used—Variable Density Random sampling and non-Cartesian Radial sampling. For the brain data, acceleration factor of 4 was used and for the other an acceleration factor of 6 was used. The reconstruction results were quantitatively evaluated based on the Normalised Mean Squared Error between the reconstructed image and the originals. The qualitative evaluation was based on the actual reconstructed images. We compared our work with four state-of-the-art parallel imaging techniques; two calibrated methods—CS SENSE and l1SPIRiT and two calibration free techniques—Distributed CS and SAKE. Our method yields better reconstruction results than all of them.

## 1. Introduction

In parallel MRI (pMRI), the object under study is scanned by multiple receiver coils. In order to expedite scanning, the K-space is partially sampled at each of the channels. The problem is to reconstruct the image given the partial K-space samples. The problem is rendered even more challenging by the fact that, each of the receiver coils has their own sensitivity profiles depending on their field of view; these sensitivity profiles are not accurately known beforehand.

In the past, all pMRI techniques required the sensitivity profile to be estimated either explicitly (SENSE [1], SMASH [2]) or implicitly (GRAPPA [3,4], SPIRiT [5]). All the aforementioned methods assume that the sensitivity maps are smooth and hence have a compact support in the Fourier domain. Thus, while acquiring the MRI scan, the centre of the K-space is densely sampled from which the sensitivity map is either explicitly estimated (SENSE or SMASH) or the interpolation weights (dependent on the sensitivity maps) are estimated (GRAPPA, SPIRiT). Unfortunately joint estimation of sensitivity maps (or related interpolation weights) is an ill-posed problem.

All the aforementioned pMRI reconstruction methods proceed in two stages—(i) In the calibration stage, the sensitivity maps or the interpolation weights are estimated; (ii) Based on these estimates, the image is reconstructed in the reconstruction stage. The reconstruction accuracy of the images is sensitive to the accuracy of the calibration stage. The calibration in turn depends on the choice of certain parameters, e.g., the window size—size of the central K-space region that has been fully sampled (for all the aforementioned methods) and the kernel size for estimating the interpolation weights (for GRAPPA and SPIRiT). These parameters are manually tuned and the best results are reported. The GRAPPA formulation has been studied in detail, and there is a study which claims to offer insights regarding the choice of GRAPPA reconstruction parameters [6]; however for other techniques such as SPIRiT and CS SENSE, there are no detailed studies on parameter tuning.

In this work, we improve upon our previous work on calibration free reconstruction (see Section 2.2). Our method reconstructs each of the different multi-coil images, which are then combined by the sum-of-squares approach (used in GRAPPA and SPIRiT). We compare our method with state-of-the-art parallel MRI reconstruction methods; two of these are calibrated techniques—CS SENSE [7] and SPIRiT and the other two are calibration free methods—DCS and SAKE. Our proposed method outperforms all of them.

Mathematically the sensitivity encoding of MR images is a modulation operation where the signal (image) is modulated by the sensitivity function (map) of the coils. All the aforesaid studies are based on the assumption the sensitivity map is smooth. Moreover the design on the receiver coils ensure that there sensitivity does not vanish anywhere, *i.e.*, there is no portion of the sensitivity map that has zeroes. This is to ensure that each of the coils collects information about the entire object under scan and no portion of the object is “invisible” to any of the coils. The sensitivity maps can thus be represented as smooth functions without any singularities. When this assumption holds, the sensitivity maps will not affect the location of the singularities/discontinuities/edges in the image. Sparsifying transforms like wavelet and finite difference, capture the discontinuities in the images, *i.e.*, the transform coefficients have high values at positions corresponding to the edges and zeros elsewhere. Since sensitivity encoding (modulation), do not affect the position of the discontinuities in the sensitivity encoded coil images, the positions of the high valued transform coefficients of the coil images will be the same for all.

Our reconstruction method is based on the fact that the position of the high valued transform coefficients in the different sensitivity encoded coil images remain the same. Based on the precepts of Compressed Sensing (CS) we formulated the reconstruction as a row-sparse Multiple Measurement Vector (MMV) recovery problem. Our method produces one sensitivity encoded image corresponding to each receiver coil in a fashion similar to GRAPPA and SPiRIT. Both of these methods reconstruct the final image as a sum-of-squares of the sensitivity encoded images. In this paper, we will follow the same combination technique.

Row-sparse MMV optimization can be either formulated as a synthesis prior or an analysis prior problem. However it is not known apriori which of these formulations will yield a better result. Even though the synthesis prior is more popular, it has been found that the analysis prior yields better results than the synthesis prior. Both of the analysis and the synthesis prior formulations can either be convex or non-convex. The Spectral Projected Gradient algorithm [8] can solve the convex synthesis prior problem efficiently. There is no efficient algorithm to solve the analysis prior problem. In the past, it has been found that for both synthesis and analysis prior, better reconstruction results can be obtained with non-convex optimization [9–11]. Following previous studies, we intend to employ non-convex optimization for solving the reconstruction problem. Since algorithms for solving such optimization problems do not exist, in this work, we derive fast but simple algorithms to solve the non-convex synthesis and analysis prior problems.

## 2. Proposed Reconstruction Technique

The K-space data acquisition model for multi-coil parallel MRI scanner is given by:

(1) $$y_{i}=F_{\Omega }x_{i}+\eta_{i},i=1\ldots C$$

where *y_i_* is the K-space data for the *i^th^* coil, *F_Ω_* is the Fourier mapping from the image space to the K-space (Ω is the set of sample points, for Cartesian sampling, *F_Ω_* can be expressed as *RF*, where *R* is a mask and *F* is the Fast Fourier Transform, but for non-Cartesian sampling, viz. Spiral, rosetta and radial, *F_Ω_* is a non-uniform Fourier transform), *x_i_* is the vectorized sensitivity encoded image (formed by row concatenation) corresponding to the *i^th^* coil, *η_i_* is the noise and *C* is the total number of receiver coils.

Since the receiver coils only partially sample the K-space, the number of K-space samples for each coil is less than the size of the image to be reconstructed. Thus, the reconstruction problem is under-determined. Following the works in CS based MR image reconstruction [12], one can reconstruct the individual coil images separately by exploiting their sparsity in some transform domain, *i.e.*, each of the images can be reconstructed by solving,

(2) $$\underset{x_{i}}{\min}{\left\|\Psi x_{i}\right\|}_{1}\text{subject to}{\left\|y_{i}-F_{\Omega }x_{i}\right\|}_{2}^{2}\leq {ɛ}_{i}$$

where Ψ is the wavelet transform *ε_i_* is the variance of noise times the number of pixels in the image.

The analysis prior optimization directly solves for the images. The synthesis prior formulation solves for the transform coefficients. In situations where the sparsifying transform is orthogonal (Orthogonal: Ψ*^T^*Ψ = I = ΨΨ*^T^*) or a tight-frame (Tight-frame: Ψ*^T^*Ψ = I ≠ ΨΨ*^T^*), the inverse problem Equation (2) can be solved via the following synthesis prior optimization:

(3) $$\underset{x_{i}}{\min}{\left\|z_{i}\right\|}_{1}\text{subject to}{\left\|y_{i}-F_{\Omega }\Psi^{T}Z_{i}\right\|}_{2}^{2}\leq {ɛ}_{i}$$

where *z_i_* = Ψ*x_i_* are the sparse transform coefficients.

However, such piecemeal reconstruction of coil images does not yield optimal results. In this paper, we will reconstruct all the coil images simultaneously by solving a MMV recovery problem. Equation (1) can be compactly represented in the MMV forms as follows:

(4) $$Y=F_{\Omega }X+N$$

where *Y* = [*y_1_*|…|*y_C_*], *X* = [*x_1_*|…|*x_C_*] and *N* = [*η_1_*|…|*η_C_*]. Here “|” denotes that the vectors are stacked as columns. In this work, we recover all the coil images *X* by solving the inverse problem Equation (4).

### 2.1. Joint Sparsity Formulation

The multi-coil images (*x_i_*'s) are formed by sensitivity encoding of the original image (to be reconstructed). All previous studies in parallel MRI assume that the sensitivity maps are smooth and have a compact support in the Fourier domain. Since the sensitivity maps are smooth, they do not alter the positions of the edges of the images although they might change the absolute values.

**Figure 1.**
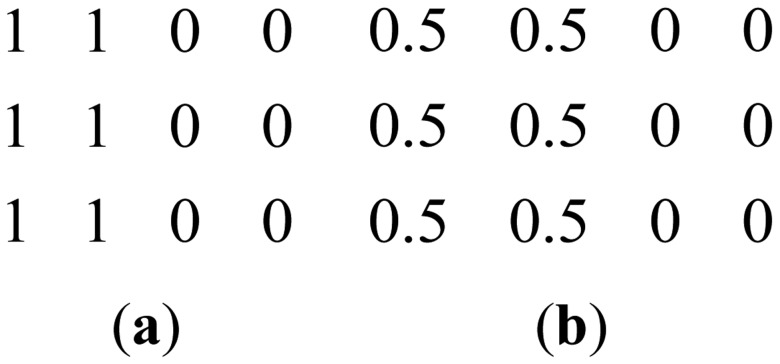
(**a**) Sharp edge and (**b**) Less prominent edge.

This can be clarified with a toy example. Figure 1a shows a prominent edge (say after sensitivity encoding by first coil) and Figure 1b shows a less prominent edge (say after sensitivity encoding by second coil).

**Figure 2.**
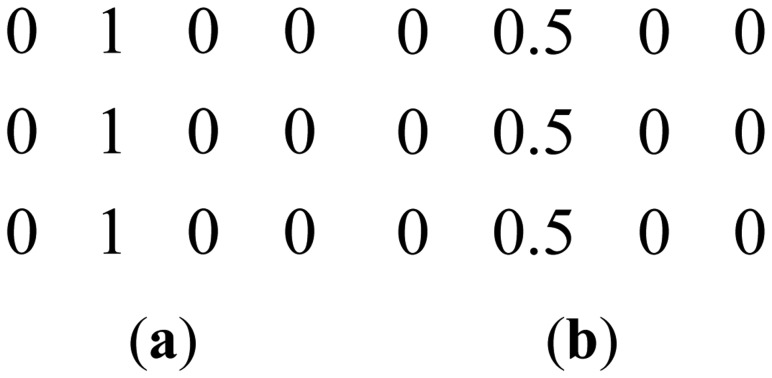
(**a**) Finite differencing of sharp edge and (**b**) Finite differencing of less prominent edge.

If finite difference is used as the sparsifying transform, the discontinuities along the edges are captured, *i.e.*, there are high values along the edges but zeroes elsewhere. The positions of the discontinuities are maintained, although the absolute values change as can be seen from Figure 2.

Based on this toy example, we consider the MMV formulation Equation (4). All the columns of *X* are images corresponding to different coils. Since the sensitivity maps of all the coils are smooth, the positions of the edges remain intact. For better clarity, we look at the images in a transform domain:

(5) $$\Psi X=Z=\left[\begin{array}{ccc} z_{1,1} & \ldots & z_{1,C}\\ \ldots & \ldots & \ldots \\ z_{r,1} & \ldots & z_{r,C}\\ \ldots & \ldots & \ldots \\ z_{n,1} & \ldots & z_{n,C} \end{array}\right]$$

where Ψ is the sparsifying transform than encodes the edges of the images, *Z* is the matrix formed by stacking the transform coefficients as columns.

In Equation (5), each row corresponds to one position. Based on the discussion so far, since the positions of the edges in the different images do not change, the positions of the high valued coefficients in the transform domain do not change either. Therefore for all the coil images the high valued transform coefficients appear at the same position. Thus the matrix *Z* is row-sparse, *i.e.*, there are a few rows with high valued coefficients while most of the rows are zeros.

We propose to solve Equation (4) by incorporating this row-sparsity information into the optimization problem. The analysis prior formulation for solving Equation (4) is as follows:

(6) $$\underset{X}{\min}{\left\|\Psi X\right\|}_{2,p}^{p}\text{subject to}{\left\|Y-F_{\Omega }X\right\|}_{F}^{2}\leq ɛ,0<p\leq 1$$

where ${\left\|Z\right\|}_{2,p}^{p}=\sum_{j=1}^{N}{\left\|Z^{j\to }\right\|}_{2}^{p}$ (*Z_j_*_→_ is the vector whose entries form the *j^th^* row of *Z* = Ψ*X*), *‖.‖_F_*denotes the Frobenius norm of the matrix and *ε* is the variance of noise multiplied by the length of the transform vector and the number of receiver coils (*C* in our case).

The values of the inner (*l_2_*) and outer (*l_p_*) norms have been suggested in [13]. The choice of such values for the norms can be understood intuitively. The inner *l_2_*-norm over the rows enforces non-zero values on all the elements of the row vector whereas the outer *l_p_*-norm enforces row-sparsity, *i.e.*, the selection of only a few rows [13].

The aforesaid problem Equation (5) is convex for *p* = 1. However, it has been found better MR image reconstruction results can be obtained if non-convex priors are used.

The analysis prior optimization directly solves for the images. The synthesis prior formulation solves for the transform coefficients. In situations where the sparsifying transform is orthogonal or a tight-frame, the inverse problem Equation (4) can be solved via the following synthesis prior optimization:

(7) $$\underset{Z}{\min}{\left\|Z\right\|}_{2,p}^{p}\text{subject to}{\left\|Y-F_{\Omega }\Psi^{T}Z\right\|}_{F}^{2}\leq ɛ,0<p\leq 1$$

where *Z* = Ψ*X*.

The images are recovered by:

(8) $$X=\Psi^{T}Z$$

The final image (*I*) is obtained from the individual coil images by sum-of-squares combination in a fashion similar to GRAPPA and SPIRiT:

(9) $$I={\left(\sum_{i=1}^{C}x_{i}^{2}\right)}^{1/2}$$

The analysis and the synthesis priors yield same results for orthogonal transforms but different results for redundant tight-frames.

### 2.2. Connection with Previous Works

In a recent work, a method similar to ours has been proposed [14]. The individual coil images were reconstructed by the solving the following optimization problem:

(10) $$\underset{X}{\min}{\left\|Y-F_{\Omega }X\right\|}_{F}^{2}+\tau {\left\|\Psi X\right\|}_{2,1}$$

This is actually the unconstrained version of our prior analysis problem Equation (6) with *p* = 1. The algorithm proposed in [14] to solve Equation (10) is ad hoc and is not derived from any optimization principle. It formulates an analysis prior problem and then suggests a synthesis prior type algorithm to solve it. Furthermore there is also the issue of choosing parameters *ε* and *τ*; For correct choice of parameters the constrained and the unconstrained versions yield the same results. Unfortunately, no analytical relationship exists between the two. It is easy to estimate *ε* since it is dependent on the noise variance. But there is no known way to estimate *τ* given the value of *ε*. One can only manually try different values of *τ* and report the best possible results. However, such a technique is not guaranteed to give optimum results in practical scenarios.

There have been other studies that used joint-sparsity models for parallel MRI reconstruction [15–17]. However, they are all modification of the basic SENSE method and require estimates of the coil sensitivities. They require explicit knowledge regarding the sensitivity maps and therefore are not calibration free techniques. The approach proposed here and those in the aforementioned studies are different.

Prior to this work, we proposed a naive version of the CaLM MRI technique [18]. There in, instead of stacking the coil images/transform coefficients as columns of a MMV matrix (as done here), were concatenated to a long vector, *i.e.*, instead of Equation (4) the data acquisition model was expressed as follows:

(11) $$y=E_{\Omega }x+\eta$$

where: $\begin{array}{ccc} y=\left[\begin{array}{c} y_{1}\\ \ldots \\ y_{C} \end{array}\right], & E_{\Omega }=\left[\begin{array}{ccc} RF_{\Omega } & 0 & 0\\ 0 & \ldots & 0\\ 0 & 0 & RF_{\Omega } \end{array}\right] & \text{and}\;x=\left[\begin{array}{c} x_{1}\\ \ldots \\ x_{C} \end{array}\right] \end{array}$

In this formulation, the vector x will be group-sparse in transform domain for the same reasons it is row-sparse in the proposed formulation. In [18] a convex group-sparse recovery problem is proposed to recover the coil images. Even though the reconstruction philosophy is the same in [18] and the proposed approach; the approach proposed in this work is more general since we can handle both convex and non-convex formulations. Also the data acquisition model Equation (4) is more natural than Equation (11).

In this work, we also do an in-depth analysis as to why the proposed technique is likely to be successful. None of the previous studies [14,18] have carried out such an analysis. Over all this work is a more generalized, thorough and in-depth extension to the prior studies.

**Figure 3.**
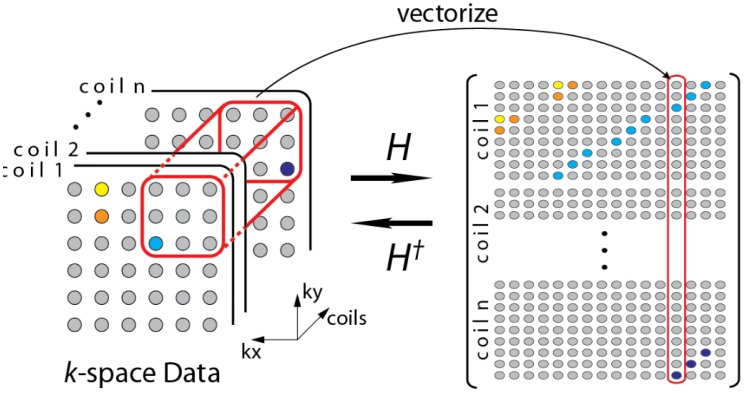
Formation of low-rank hankel matrix.

During the review, one of the reviewers pointed out to a few recent studies that do not require a calibration stage [19,20]. The formulation in [19] can be understood from the following diagram (Figure 3)—overlapping blocks from all the channels are vectorized and stacked as columns of a Hankel matrix *A*.

The Hankel matrix thus formed is low-rank owing to local correlations. In [18] the low-rank structure is exploited to recover the coil images. This is a good intuitive approach, but the main problem with this approach is that the Hankel matrix thus formed is huge owing to overlap of the blocks. Estimating the low-rank matrix is an iterative process and at each iteration the SVD of this matrix needs to be computed. Computing the SVD for such a large matrix becomes infeasible in practice. This (low-rank) assumption (behind SAKE) follows from inuition but is not very practical for large scale problems especially for 3D volume reconstruction.

SAKE is pegged on the idea that the coil images are correlated spatially; also the various channel images are correlated. Thus, the K-space samples are also correlated (The Fourier transform being orthogonal do not disturb the correlation). To overcome the computational issue of SAKE, the CLEAR technique was proposed in [20]. In CLEAR, a partial Hankel matrix is formed by considering a small section of the K-space. CLEAR assumes that the K-space is correlated locally. However, such an assumption does not follow readily from the mathematics of MRI acquisition—local correlation in the pixel domain does not translate to local correlation in the Fourier frequency domain. Thus, although CLEAR addresses the computational issues of SAKE, it introduces more severe problems—the reconstruction shows heavy artifacts owing to incorrect modeling.

## 3. Theoretical Understanding of Proposed Approach

A lot of practical CS problems exploit the sparsity of the natural signals in the wavelet basis in order to reconstruct them. The sparsity of the wavelet coefficients arises on account of the piecewise smooth (e.g., piecewise polynomial) structure of such signals, and the vanishing moments of wavelets. A precise way of describing this is that the action of any wavelet *ψ*(*t*) can be regarded as a “smoothed” derivative operation [21], namely:

(12) $$\int f(t)\psi (t-t_{0})dt=D^{\left(n\right)}(f*\phi )(t_{0})$$

where the order of differentiation *n* is precisely the number of vanishing moments of *ψ*(*t*). Here *φ*(*t*) is some low-pass function matched with the wavelet *ψ*(*t*). As a result, large wavelet coefficients are obtained in the vicinity of singularities, while a relatively smaller response is obtained in the smooth portions of the signal.

In this sub-section, we make some observations on how the sparsity of the piecewise smooth signal is affected by modulation. To keep it simple, we work with one-dimensional signals. Let *f*(*t*) be a piecewise smooth signal that is multiplied by a waveform *m*(*t*) to get the modulated signal *g*(*t*) = *f*(*t*)*m*(*t*). A natural question then is whether *g*(*t*) is sparse in the wavelet domain, and if so, does it have the same sparsity signature as *f*(*t*)? By sparsity signature, we simply mean the set of points where the wavelet coefficients are larger than some threshold. The actual size of the response could, however, be very different. Simulation results confirm that this is indeed the case, provided that *m*(*t*) and some of its derivatives is non-vanishing. These observations can be explained more precisely.

Note that if *f*(*t*) and *g*(*t*) are singular at the same set of points, then they clearly have the same sparsity signature. The questions then is can the modulation operation create new discontinuities or erase some of the existing ones? It is clear that *g*(*t*) cannot have a discontinuity if both *f*(*t*) and *m*(*t*) are smooth.

Therefore, the only situation of interest is that in which *f*(*t*) has a discontinuity and we ask as to under what conditions on *m*(*t*) will *g*(*t*) exhibit a discontinuity? For the simplest case of *jump discontinuity*, we easily see the following.

**Figure 4.**
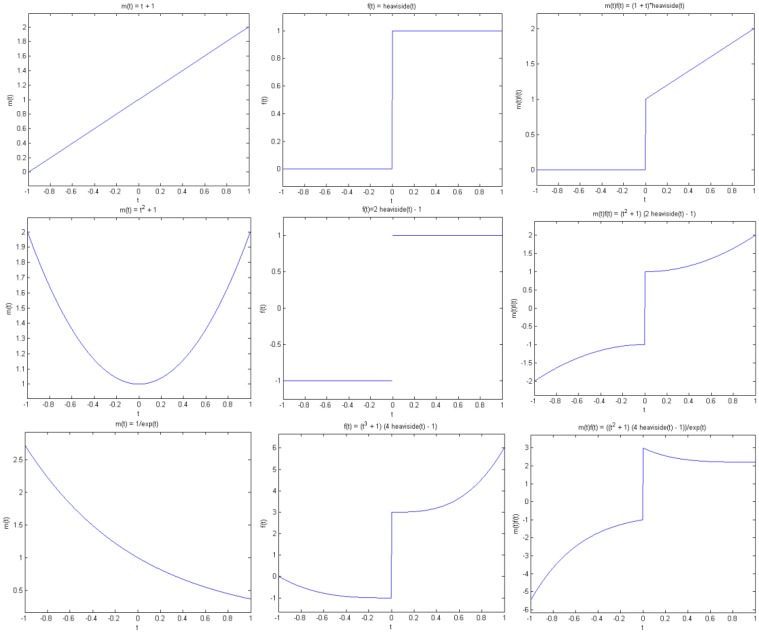
(**Left**)—Modulation function *m*(*t*); (**Middle**)—Signal *f*(*t*); (**Right**)—Modulated signal *m*(*t*)*f*(*t*).

**Proposition 1** (Jump singularity). Suppose *f*(*t*) has a jump discontinuity at *t* = *t*_0_, and *m*(*t*) is smooth. Then *g*(*t*) has a jump at *t*_0_ if and only if *m*(*t*_0_) is non-zero (see Figure. 4).

Note that by smooth we mean that *m*(*t*) is continuous and has sufficient derivatives. On the other hand, *f*(*t*) has a jump at *t*_0_ in the sense that *f*(*t*) is smooth away from *t*_0_, but has different left and right limits at *t*_0_, that is, *f*(*t*) tends to different values as *t* approaches *t*_0_ from the left and right of *t*_0_. As a simple example, consider the Heaviside function with a transition at *t*_0_.

In practice this proposition demands that the sensitivity map (modulation function) should be smooth and non-vanishing. The fact that the sensitivity map is smooth is well known and is the basis of all studies in parallel MRI. But we make the additional demand that the sensitivity map should be non-vanishing as well. Ideally this constraint is satisfied by the design of the scanner—there is no portion of the subject which is completely blind to a particular channel; thus the sensitivity profile for all the channels are non-vanishing.

Note that higher-order singularities can arise when two smooth functions are glued together. For example, consider the function obtained by gluing the zero function and a polynomial:

(13) $$f(t)=\{\begin{array}{ccc} 0 & if & t\leq 0\\ x^{n} & if & t>0 \end{array}$$

It is clear that *f*(*t*) is continuous. In fact, *f*(*t*) has n derivatives. However, the *n*-th derivative *f*^(^*^n^*^)^(*t*) has a jump at *t*_0_. As a result, the wavelet transform of *f*(*t*), obtained using a wavelet with sufficient vanishing moments, is sparse with a large non-zero response around *t*_0_.

So what is the effect of modulation on the wavelet transform of such signals? Of course, one would expect *g*(*t*) to have at most *n* derivatives. The only way it could have more derivatives is if the corresponding derivatives of *m*(*t*) vanish at *t*_0_.

**Proposition 2** (Higher-order singularity). Suppose *f*(*t*) has *n* derivatives at *t* = *t*_0_, but its *n*-th derivative is discontinuous at *t*_0_. Then *g*(*t*) can have *m > n* derivatives at *t*_0_ if and only if *m*^(^*^k^*^)^(*t*_0_) = 0 for *n* ≤ *k* ≤ *m* − 1. Otherwise, the *g*(*t*) would have at most *n* derivatives at *t*_0_.

**Figure 5.**
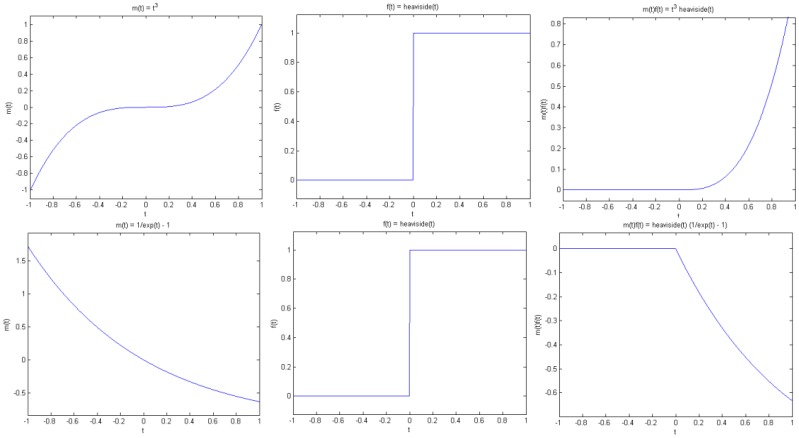
(**Left**)—Modulation function *m*(*t*); (**Middle**)—Signal *f*(*t*); (**Right**)—Modulated signal *m*(*t*)*f*(*t*).

Combined with Equation (12), the implication of this observation is that if the wavelet at least *n* vanishing moments, then the wavelet transforms of both *f*(*t*) and *g*(*t*) would exhibit a large response around *t*_0_, unless the *n*-th and larger derivatives of *m*(*t*) are zero at *t*_0_ (see Figures 4 and 5). In summary, if it can be guaranteed that *m*(*t*) and its derivatives are always positive (or negative), then the wavelet coefficients of *g*(*t*) would have the same sparsity signature as that of *f*(*t*).

For parallel MRI reconstruction, the sensitivity map modulates the underlying signal (MR image). The sensitivity maps are assumed to be smooth and can be modeled as polynomials [22]. The design of the scanner ensures that there are no singularities in the sensitivity maps; physically this ensures that each receiver coil has information about the full image. Based on the discussion in this sub-section, this guarantees that the jump discontinuities in the MR image are preserved after sensitivity encoding. Hence, the positions of the high valued wavelet transform coefficients will remain unchanged before and after sensitivity encoding.

We show a toy example. We considered a function *f*(*t*) = (1 + *t*^2^) (2 heaviside(t)-4 heaviside(t-T)). Which was modulated by two polynomials of small order; the modulation functions are:

$$\begin{array}{l} m1(t)=t^{3}+t^{2}+1\\ m2(t)=t^{2}+1 \end{array}$$

**Figure 6.**
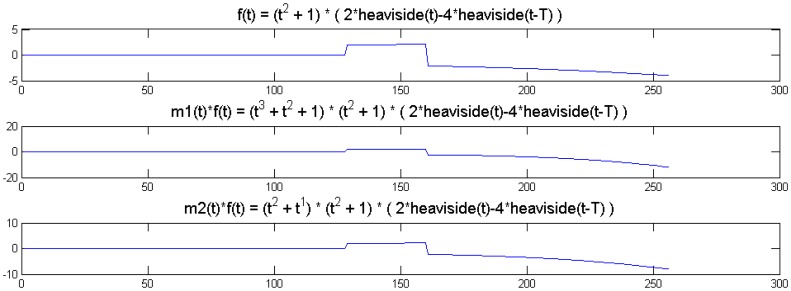
Original and modulated signals. (**Top**) to (**bottom**): *f*(*t*); *m1*(*t*) × *f*(*t*); *m2*(*t*) × *f*(*t*).

The original function and its modulated versions are shown in Figure 6.

**Figure 7.**
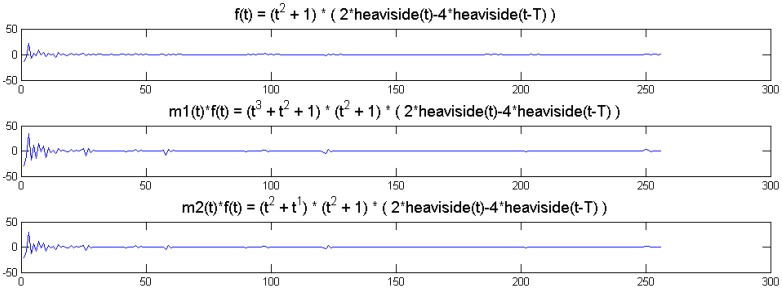

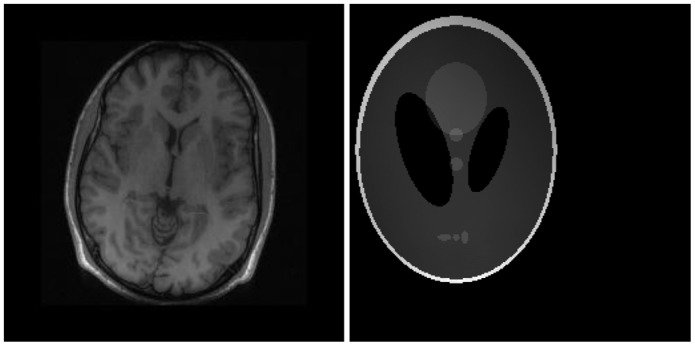
Wavelet transform of original and modulated signals. (**Top**) to (**bottom**): *f*(*t*); *m1*(*t*) × *f*(*t*); *m2*(*t*) × *f*(*t*). Groundtruth images: Brain and Shepp-Logan Phantom.

We compute the wavelet transforms of the original and the modulated signals. These are shown in Figure 7. Daubechies wavelets of order 16 is used and the decomposition scale is 7.

It can be seen from Figure 6 that the sparsity signatures are exactly the same. The wavelet transform of the original and the modulated signals have high valued coefficients at the same positions; but the actual values at these positions are varying.

## 4. Optimization Algorithms

The Majorization-Minimization (MM) approach [23] is employed to derive solution to the following problems:

(14a) $${\begin{array}{cc} \text{Synthesis}:\underset{X}{\text{min}}{\left\|X\right\|}_{2,p}^{p} & \text{subject to}\left\|Y-HX\right\| \end{array}}_{F}^{2}\leq ɛ$$

(14b) $${\begin{array}{cc} \text{Analysis}:\underset{X}{\text{min}}{\left\|AX\right\|}_{2,p}^{p} & \text{subject to}\left\|Y-HX\right\| \end{array}}_{F}^{2}\leq ɛ$$

For the synthesis prior *X* = *Z*, *H* = *F_Ω_*Ψ and for analysis prior, *A* = Ψ and *H* = *F_Ω_*.

Instead of solving the aforesaid constrained problems, we propose solving their unconstrained counterparts,

(15a) $$\underset{X}{\min}J_{1}(X),\text{where}\;J_{1}(X)=\frac{1}{2}{\left\|Y-HX\right\|}_{F}^{2}+\lambda {\left\|X\right\|}_{2,p}^{p}$$

(15b) $$\underset{X}{\min}J_{2}(X),\text{where}\;J_{2}(X)=\frac{1}{2}{\left\|Y-HX\right\|}_{F}^{2}+\lambda {\left\|AX\right\|}_{2,p}^{p}$$

The constrained and the unconstrained formulations are equivalent for proper choice of the Lagrangian *λ*. Unfortunately for most practical problems it is not possible to determine *λ* explicitly by analytical means. Therefore, instead of ‘guessing’ *λ*, given the value of *ε* (as in [14]), we will reach the solution of the constrained problem by iteratively solving a series of unconstrained problems with decreasing values of *λ*. Such cooling techniques are successful since the Pareto curve for the said problem is smooth [24]; similar cooling algorithms have been successfully used in the past for solving Compressed Sensing problems [24–26].

### 4.1. Solving the Unconstrained Problems

We solve this problem by the Majorization-Minimization (MM) approach [23]. The generic MM algorithm is as follows,

Let *J*(*x*) be the (scalar) function to be minimized

1. Set *k* = 0 and initialize *x_0_*.
  Repeat step 2–4 until suitable a stopping criterion is met.
2. Choose *G_k_*(*x*) such that
  - *G_k_*(*x*) ≥ *J*(*x*) for all *x*.
  - *G_k_*(*x_k_*) = *J*(*x_k_*).
3. Set *x_k +_*_1_ as the minimizer for *G_k_*(*x*).
4. Set *k* = *k +* 1, go to step 2.

For this paper, the problems to be solved are Equations (15a) and (15b). They do not have a closed form solution and therefore must be solved iteratively. At each iteration, we chose

(16a) $$G_{1}^{\left(k\right)}(x)={\left(X-X^{\left(k\right)}\right)}^{t}(\alpha I-H^{T}H)(X-X^{\left(k\right)})+{\left\|Y-HX\right\|}_{F}^{2}+\lambda {\left\|X\right\|}_{2,p}^{p}$$

(16b) $$G_{2}^{\left(k\right)}(x)={\left(X-X^{\left(k\right)}\right)}^{t}(\alpha I-H^{T}H)(X-X^{\left(k\right)})+{\left\|Y-HX\right\|}_{F}^{2}+\lambda {\left\|AX\right\|}_{2,p}^{p}$$

$G_{1}^{\left(k\right)}(x)$ and $G_{2}^{\left(k\right)}(x)$ satisfies the condition for MM algorithm when *α* ≥ max *eigvalue*(*H^T^H*).

Equations (16a) and (16b) can alternately be expressed as,

(17a) $$G_{1}^{\left(k\right)}(x)=\alpha {\left\|X^{\left(k\right)}+\frac{1}{\alpha }H^{T}(Y-HX)-X\right\|}_{2}^{2}+\lambda {\left\|X\right\|}_{2,p}^{p}+K_{1}$$

(17b) $$G_{1}^{\left(k\right)}(x)=\alpha {\left\|X^{\left(k\right)}+\frac{1}{\alpha }H^{T}(Y-HX)-X\right\|}_{2}^{2}+\lambda {\left\|AX\right\|}_{2,p}^{p}+K_{1}$$

where *K_1_* and *K_2_* are terms independent of *X*.

Minimizing Equations (17a) and (17b) are the same as the following,

(18a) $$\underset{X}{\min}G_{1}^{\left(k\right)}(X),G_{1}^{\left(k\right)}(X)=\frac{1}{2}{\left\|B^{\left(k\right)}-X\right\|}_{F}^{2}+\frac{\lambda }{\alpha }{\left\|X\right\|}_{2,p}$$

(18b) $$\underset{X}{\min}G_{2}^{\left(k\right)}(X),G_{2}^{\left(k\right)}(X)=\frac{1}{2}{\left\|B^{\left(k\right)}-X\right\|}_{F}^{2}+\frac{\lambda }{\alpha }{\left\|AX\right\|}_{2,p}$$

where $B^{\left(k\right)}=X^{\left(k\right)}+\frac{1}{\alpha }H^{T}(Y-HX^{\left(k\right)})$.

These updates Equation (18) are known as the Landweber iterations.

For the synthesis prior problem, we need to solve Equation (18a) at each iteration. Taking the derivative of $G_{1}^{\left(k\right)}(X)$ we get,

$$\frac{dG_{1}^{\left(k\right)}(X)}{dX}=X-B^{\left(k\right)}+\frac{\lambda }{\alpha }\Lambda \text{signum}(X)$$

where signum is the sign of the components in *X*, where $\Lambda =\text{diag}({\left\|X^{(k)j\to }\right\|}_{2}^{p-2})\left|X^{\left(k\right)}\right|$; here ${\left\|X^{(k)j\to }\right\|}_{2}^{p-2}$ means that the *l_2_*-norm of the *j^th^* row of *X* is raised to power *p-2*.

Setting the derivative to zero and re-arranging, we get:

(19) $$B=X+\frac{\lambda }{\alpha }\Lambda \text{signum}(X)$$

This can be solved by the following soft-thresholding:

(20) $$X^{\left(k+1\right)}=\text{signum}(B^{\left(k\right)})\max(0,\left|B^{\left(k\right)}\right|-\frac{\lambda }{\alpha }\Lambda )$$

Equations (18a) and (20) suggest a compact solution for the unconstrained synthesis prior problem. This is given in the following algorithm.



**Algorithm 1:** Unconstrained Synthesis Prior
Initialize:*X*^(0)^**=** 0 Repeat until convergence:
$\begin{array}{c} B^{\left(k\right)}=X^{\left(k\right)}+\frac{1}{\alpha }H^{T}(Y-HX^{\left(k\right)})\\ X^{\left(k+1\right)}=\text{signum}(B^{\left(k\right)})\max(0,\left|B^{\left(k\right)}\right|-\frac{\lambda }{\alpha }\Lambda ) \end{array}$


Solving the analysis prior problem requires minimization of Equation (18b) in each iteration. Taking the derivative of $G_{2}^{\left(i\right)}(X)$ $G_{2}^{\left(i\right)}(X)$ we get:

(21) $$\frac{dG_{2}^{\left(k\right)}(X)}{dX}=X-B^{\left(i\right)}+\frac{\lambda }{\alpha }A^{T}\Omega AX$$

where $\Omega =\text{diag}({\left\|W^{(i)j\to }\right\|}_{2}^{p-2})\Omega =\text{diag}({\left\|W^{(i)j\to }\right\|}_{2}^{p-2})$ and *W* = *AX*.

Setting the gradient to zero we get:

(22) $$(I+\frac{\lambda }{\alpha }A^{T}\Omega A)X=B^{\left(k\right)}$$

It is not possible to solve Equation (22) directly as the sparsifying transform (*A*) in most cases is available as a fast operator and not as an explicit matrix. To overcome this problem, the matrix inversion lemma is used to simplify it:

$${\left(I+\frac{\lambda }{\alpha }A^{T}\Omega A\right)}^{-1}=I-A^{T}{\left(\frac{\alpha }{\lambda }\Omega^{-1}+AA^{T}\right)}^{-1}A$$

From Equation (22), we have using the above identity:

$$X=B^{\left(k\right)}-A^{T}{\left(\frac{\alpha }{\lambda }\Omega^{-1}+AA^{T}\right)}^{-1}AB^{\left(k\right)}$$

Adding *cz* to both sides and subtracting *AA^T^z* from both sides gives the equivalent equation we get:

(23) $$z^{\left(k+1\right)}={\left(\frac{\alpha }{\lambda }\Omega^{-1}+cI\right)}^{-1}(cZ^{\left(k\right)}+A(B^{\left(k\right)}-A^{T}Z^{\left(k\right)}))$$

(24) $$X^{\left(k+1\right)}=B^{\left(k\right)}-A^{T}Z^{\left(k\right)}$$

where *c* ≥ max *eigvalue*(*A^T^A*).

This leads to the following algorithm for solving the analysis prior joint-sparse optimization problem.


**Algorithm 2:** Unconstrained Analysis Prior
Initialize:*X*^(0)^=0 Repeat until convergence:
$\begin{array}{c} B^{\left(i\right)}=X^{\left(i\right)}+\frac{1}{\alpha }H^{T}(Y-HX^{\left(i\right)})\\ Z^{\left(i+1\right)}={\left(\frac{\alpha }{\lambda }\Omega^{-1}+cI\right)}^{-1}(cZ^{\left(i\right)}+A(B^{\left(i\right)}-A^{T}Z^{\left(i\right)}))\\ X^{\left(i+1\right)}=B^{\left(i\right)}-A^{T}Z^{\left(i\right)} \end{array}$


### 4.2. Solving the Constrained Problem via Cooling

We have derived algorithms to solve the unconstrained problems. As mentioned before, the constrained and the unconstrained forms are equivalent for proper choice of *ε* and *λ*. However, there is no analytical relationship between them in general. When faced with a similar situation, we employed the cooling technique following previous studies [24–26].

The cooling technique solves the constrained problem in two loops. The outer loop decreases the value of *λ*. The inner loop solves the unconstrained problem for a specific value *λ*. As *λ* is progressively decreased, the solution of the unconstrained problem reaches the desired solution. Such a cooling technique works because the pareto curve between the objective function and the constraint is smooth. The cooling algorithm for the synthesis and analysis prior are:

**Algorithm 3:** Synthesis Prior Algorithm
Initialize:*X*^(0)^ =0; *λ* < max(*P^T^x*) Choose a decrease factor (*DecFac*) for cooling *λ* Outer Loop: While^1^
${\left\|y-Hx\right\|}_{F}^{2}\geq ɛ$Inner Loop: While^2^
$\begin{array}{c} \frac{J^{\left(i\right)}-J^{\left(i+1\right)}}{J^{\left(i\right)}+J^{\left(i+1\right)}}\geq \text{Tol}\\ J^{\left(i\right)}={\left\|Y-HX^{\left(i\right)}\right\|}_{F}^{2}+\lambda {\left\|X^{\left(i\right)}\right\|}_{2,p}^{p} \end{array}$Compute:
$B^{\left(i\right)}=X^{\left(i\right)}+\frac{1}{\alpha }H^{T}(Y-HX^{\left(i\right)})$Compute:
$\begin{array}{c} X^{\left(i+1\right)}=\text{signum}(B^{\left(i\right)})\max(0,\left|B^{\left(i\right)}\right|-\frac{\lambda }{\alpha }\Lambda )\\ J^{\left(i+1\right)}={\left\|Y-HX^{\left(i\right)}\right\|}_{F}^{2}+\lambda {\left\|X^{\left(i\right)}\right\|}_{2,p}^{p} \end{array}$End While^2^ (inner loop ends)*λ* = *λ* x *DecFac*End While^1^ (outer loop ends)


**Algorithm 4:** Analysis Prior Algorithm
Initialize:*X*^(0)^=0; *λ* < max(*P^T^x*)Choose a decrease factor (*DecFac*) for cooling *λ*Outer Loop: While^1^
${\left\|y-Hx\right\|}_{F}^{2}\geq ɛ$Inner Loop: While^2^
$\begin{array}{c} \frac{J^{\left(k\right)}-J^{\left(k+1\right)}}{J^{\left(k\right)}+J^{\left(k+1\right)}}\geq \text{Tol}\\ J^{\left(k\right)}={\left\|Y-HX^{\left(k\right)}\right\|}_{F}^{2}+\lambda {\left\|AX^{\left(k\right)}\right\|}_{2,p}^{p} \end{array}$Compute:
$B^{\left(k\right)}=X^{\left(k\right)}+\frac{1}{\alpha }H^{T}(Y-HX^{\left(k\right)})$Update:
$Z^{\left(k+1\right)}={\left(\frac{\alpha }{\lambda }\Omega^{-1}+cI\right)}^{-1}(cZ^{\left(k\right)}+A(B^{\left(k\right)}-A^{T}Z^{\left(k\right)}))$Update:
$\begin{array}{c} X^{\left(K+1\right)}=B^{\left(i\right)}-A^{T}Z^{\left(k\right)}\\ J^{\left(k+1\right)}={\left\|Y-HX^{\left(k\right)}\right\|}_{F}^{2}+\lambda {\left\|AX^{\left(k\right)}\right\|}_{2,p}^{p} \end{array}$End While^2^ (inner loop ends)*λ* = *λ* × *DecFac*End While^1^ (outer loop ends)


In this work, we proposed solving the reconstruction problem via non-convex optimization algorithms. Theoretically one may argue about the convergence of such algorithms to local minima. However, in practice it has never been a problem. In previous studies [9–11,27], non-convexity never posed to be problem for MRI reconstruction.

## 5. Experimental Evaluation

There are two sets of ground-truth data used for our experimental evaluation (Figure 7). The brain data and the Shepp-Logan phantom have been used previously in [4]. The brain data is a fully sampled T1 weighted scan of a healthy volunteer. The volunteer was scanned using Spoiled Gradient Echo sequence with the following parameters—echo time = 8 ms; repetition time = 17.6 ms; flip angle = 20 degrees. The scan was performed on a GE Sigma-Excite 1.5-T scanner, using an eight-channel receiver coil. The 8-channel data for Shepp-Logan phantom was simulated. The ground-truth is formed by sum-of-squares reconstruction of the multi-channel images.

**Figure 8.**
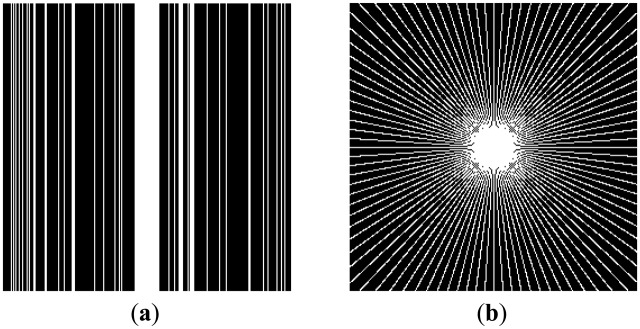
(**a**) VD Random Sampling, (**b**) Radial sampling.

In this work, we show results for two different K-space sampling schemes (Figure 8)—Variable Density Random Sampling (Cartesian) and Radial Sampling (non-Cartesian). In VD Random (VDR) Sampling, the center of the K-space is densely sampled, while the rest of the K-space is sparsely sampled by randomly omitting lines in the phase encoding direction. This is widely used sampling method for parallel MRI. Radial sampling is one of the fastest sampling methods [28,29] and has been previously used in parallel MRI [30]. For the brain image, the acceleration factor of 4 is used, for the Shepp-Logan phantom, acceleration factor of 6 is used for both Variable Density random sampling and radial sampling.

We compare our proposed method with two state-of-the art calibrated methods—L1SPIRiT [4] (frequency domain method) and CS SENSE [6] (image domain method) and two calibration free techniques DCS [14] and SAKE [19]. For our proposed method, the mapping from non-Cartesian K-space to the Cartesian image space is the Non-Uniform Fast Fourier Transform (NUFFT) [31,32].

For CS SENSE the sensitivity profiles are estimated in the fashion shown in [30]. A Kaiser-Bessel window at the center of the K-space is densely sampled, from which a low resolution image for each coil is obtained. These images are combined by sum-of-squares. The sensitivity map is computed by dividing the low resolution image of the corresponding coil by the combined sum-of-squares image.

Our proposed method and the DCS based method propounded in [14] do not require any parameter estimation. In [14], the reconstruction is solved via Equation (10). However, as mentioned earlier, it is not possible to determine the parameter τ analytically. For this work, we determine the value of *τ* as specified in [14]—1/500 of the maximum (in absolute value) of the zero-filled image for the first 50 iterations, and 1/100 of the maximum value for the last 10 iterations. Sixty iterations were used to generate the final image.

For our non-convex formulation, we found that the best results were obtained for *p* = 0.5 (this value of p has also been suggested in [11]). The quantitative reconstruction results are shown in Table 1. Normalized Mean Squared Error (NMSE) is the metric used for evaluation. The best reconstruction (lowest error) results are shown in bold.

The DCS reconstruction yields the worse results. This is expected—DCS is an ad hoc algorithm and consequently it fails. Our proposed non-convex analysis prior formulation yields the best results. The synthesis prior formulation is slightly worse off than the analysis prior. The SAKE technique does not yield as good results as our proposed technique. CS SENSE and l1SPIRiT yield better results than SAKE, but they have to be thoroughly calibrated and hence are not robust.

**Figure 9.**
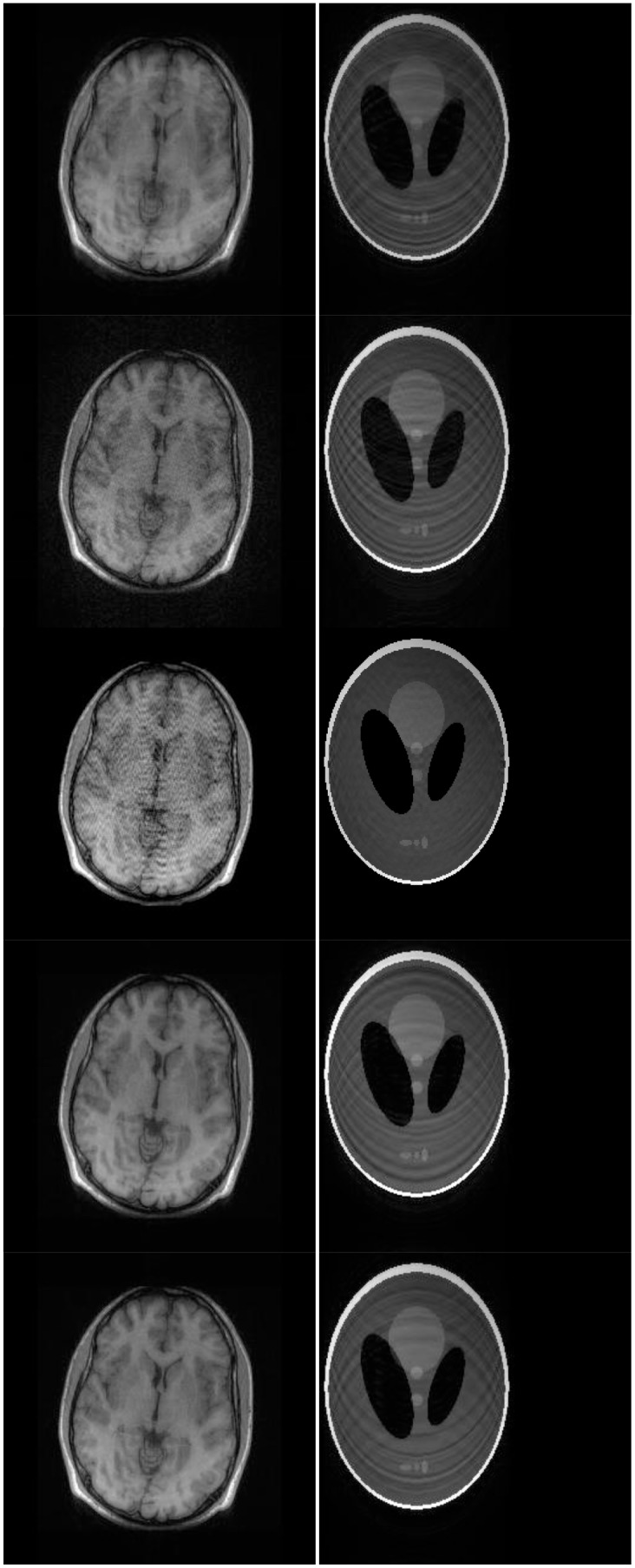

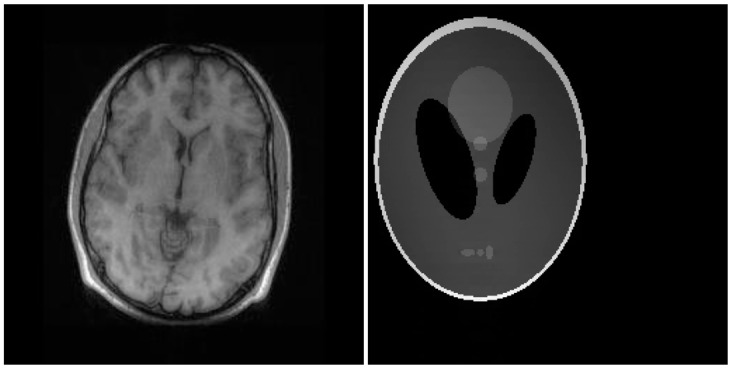
Reconstruction for Variable Density Random sampling. From Top to Bottom: DCS Reconstruction, l1SPIRiT, CS SENSE, SAKE, Proposed Non-Convex Synthesis Prior, Proposed Non-Convex Analysis Prior.

Although NMSE is an often used metric for evaluating the reconstruction accuracy, it does not always reflect the qualitative aspects of reconstruction. For qualitative evaluation we show the reconstructed images in Figure 9. Owing to limitations in space we only show the results for variable density random sampling. The qualitative results more or less corroborate the quantitative results. With 6-fold undersampling, all the methods apart from our proposed analysis prior formulation yields significant reconstruction artifacts.

**Figure 10.**
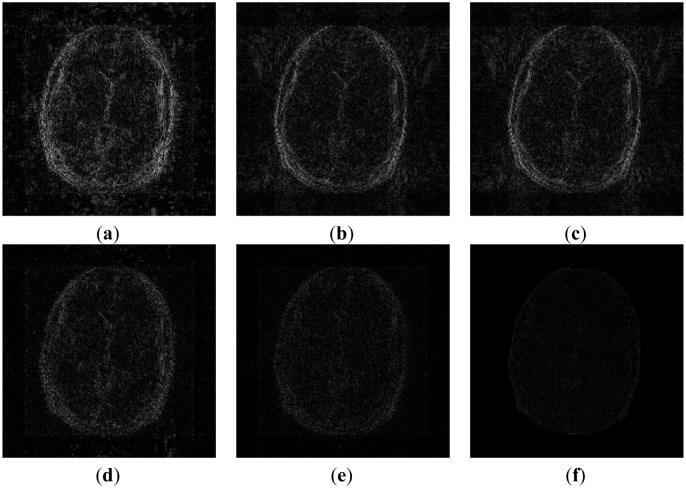
Difference Images. (**a**) DCS; (**b**) CS SENSE; (**c**) SAKE; (**d**) l1SPIRiT; (**e**) non-convex synthesis prior; (**f**) non-convex analysis prior.

In order to elucidate the reconstruction even more, we show the difference (between groundtruth and reconstructed) images for the brain image. The difference images are shown in Figure 10. The contrast of the difference images have been enhances five times for visual clarity. The difference images corroborate our previous findings. We see that the DCS reconstruction yields the worst results. CS SENSE and SAKE yields almost similar difference images; l1SPIRiT slightly improves upon CS SENSE and SAKE. Our analysis prior formulation yields the best results; the synthesis prior is better than l1SPIRiT bust is slightly worse than the analysis prior.

## 6. Conclusions

State-of-the-art parallel MRI techniques either implicitly or explicitly require a calibration stage to estimate the sensitivity maps (for SENSE, SMASH and related techniques) or interpolation weights (for GRAPPA, SPIRiT and related techniques). Thus, all these methods are sensitive to the calibration stage. In recent times there is a concerted effort in developing calibration free reconstruction techniques. In this paper we improve upon a previous technique calibration free reconstruction technique [18].

We compare our proposed technique with other calibrated and calibration free methods. We find that our proposed non-convex analysis prior formulation always yields the best results. However there are two shortcomings with the proposed method. The first one is more of a constraint than a shortcoming. Our technique does not work with uniform periodic undersampling. This is because, our solution approach requires solving an under-determined problem Equation (4) and is based on the tenets of Compressed Sensing; and Compressed Sensing demands that the sampling scheme should be randomized.

The second problem with our work is on the assumption that the modulation function is smooth that does not change the number of discontinuities in the image. However, the function can introduce new discontinuities if the function is zero in certain positions. Ideally this is taken care of during the design of the scanner, the FOV is designed such that no area of the subject is completely blind to the channel. However, if the SNR the modulation function can be effectively zero. This would violate the row-sparsity assumption and our method would fail to produce good results.

## Figures and Tables

**Table 1.** Comparison of reconstruction accuracies for calibration-free techniques.

**Image →**	**Brain**		**Phantom**	

Type of Sampling →	VDR	Radial	VDR	Radial
l1SPIRiT [4]	0.13	0.07	**0.13**	0.09
CS SENSE [5]	0.16	0.28	0.14	0.04
DCS reconstruction [13]	0.25	0.19	0.29	0.17
SAKE [19]	0.14	0.14	**0.13**	0.10
Proposed non-convex synthesis prior	0.08	**0.03**	0.15	0.01
Proposed non-convex analysis prior	**0.06**	**0.03**	**0.13**	**0.00**
